# Around the Hospitals

**Published:** 1893-09-09

**Authors:** 


### Around the Hospitals.

Notice to the Managers of Institutions.—Special space is now reserved for the insertion of notices of hospital meetings and festivals. The Editor requests that all notices may reach The Hospital Office, 428, Strand, at least one week before the date of each meeting.

**North Devon Infirmary.**—The annual report of this hospital is scarcely as satisfactory as could be wished. The committee regret to show a balance of £340 17s. 7d. on the wrong side, and make an urgent appeal for increased support. While subscriptions and donations have sadly diminished, the expenditure for the past year has amounted to £2,439 15s. 10d., as against £2,290 4s. 4d. for 1891, and the number of both out-patients and in-patients has increased. The committee "have much pleasure in bearing their testimony to the efficient working of the institution," and the various officers receive due recognition for the able manner in which they have discharged their duties, to the entire satisfaction of the committee. Earl Fortescue, who presided at the annual meeting, attributed some of the falling off in the funds to the increasing number of cottage hospitals, which, while most necessary in such places as Lynton, cut off from railway communication, yet were no substitute for the larger institutions, and undoubtedly directed money from the support of the latter. The committee report that the convalescent home in connection with the infirmary at Morthoe is being put into perfect order, and is proving of very great value. The institution has sustained the loss of a kind friend in the death of Mr. William Avery, whose warm interest in its affairs was always to be relied on. We trust the funds which are needed to carry on the useful work of the North Devon Infirmary will be speedily forthcoming.

**The Lewes Dispensary.**—The annual general meeting of the governors and subscribers of the Victoria Hospital and Dispensary, Lewes, saw the adoption of a satisfactory report. The income for the past year, amounted to £818, and the expenditure to £732, showing a balance in favour of the hospital. Much help had been received from the Hospital Sunday and Saturday Funds, which the committee gratefully acknowledged. The Mayor and Mayoress received the best thanks of the directors for the valuable assistance they had rendered to the institution, and a warm vote of thanks was also accorded to the medical staff for their services during the past year. Much regret was expressed at the resignation of the resident medical officer, Mr. Charles Ryall. It is encouraging amid the reports from so many charitable institutions showing decreased subscriptions, to find one which tells a more cheerful tale, and the evidently harmonious spirit which characterises the relations between this hospital and dispensary and the Lewes people reflects great credit on all concerned. We sincerely hope an equally satisfactory report may be forthcoming next year, and that help will continue to flow in.

